# Association Between Screen Exposure and Insomnia Among Patients With Breast Cancer: Cross-Sectional Study

**DOI:** 10.2196/85837

**Published:** 2026-07-23

**Authors:** Lumin Liu, Ming Yang, Meina Ye, Junjie Anderson Lu, Yiyue Dong, Shunxian Zhang, Sandra Teresa Chow, Junwei Hu, Shihan Jiang, Xi Wang, Anran Cheng, Yisheng Huai, Zhicheng Guo, Qian Fan, Yuelai Chen, Ping Yin

**Affiliations:** 1 Sleep Medicine Center, Longhua Hospital Shanghai University of Traditional Chinese Medicine, 725 South Wanping Road, Shanghai, 200032, China, 86 18917561621; 2 Clinical Research Center, Longhua Hospital Shanghai University of Traditional Chinese Medicine, Shanghai, China; 3 Department of Breast Surgery, Longhua Hospital Shanghai University of Traditional Chinese Medicine, Shanghai, China; 4 Department of Epidemiology and Population Health, School of Medicine, Stanford University, Stanford, CA, United States

**Keywords:** screen exposure, insomnia, breast cancer, perceived stigma, cross-sectional

## Abstract

**Background:**

Insomnia burdens patients with breast cancer, exceeding its prevalence in other malignancies. While excessive screen time is known to disrupt sleep in children and adolescents, its impact on patients with breast cancer remains underexplored, particularly regarding how perceived stigma may modify screen-related sleep disruption.

**Objective:**

This study aimed to investigate the associations between both objectively measured total daily screen time and self-reported presleep screen time and insomnia in patients with breast cancer while assessing the effect measure modification by perceived stigma.

**Methods:**

This cross-sectional study recruited 778 patients with breast cancer from the Department of Breast Surgery, Longhua Hospital, Shanghai University of Traditional Chinese Medicine, between August 1, 2023, and February 20, 2024. Total daily and presleep screen time were assessed as exposures, whereas insomnia, diagnosed using a standardized clinical assessment based on *Diagnostic and Statistical Manual of Mental Disorders, Fifth Edition*, criteria, served as the main outcome. Multivariable logistic regression models were used to estimate odds ratios and 95% CIs for the associations between screen time and insomnia using hierarchical adjustment across 3 models.

**Results:**

Of the 240 participants with complete data on total daily screen time (mean age 51.82, SD 10.62 years), 186 (77.5%) were diagnosed with insomnia. Of the 644 participants with data on presleep screen time (mean age 53.46, SD 10.76 years), 419 (65.1%) met the criteria for insomnia. The median total daily screen time was 334.00 (IQR 232.50-440.25) minutes, whereas the median presleep screen time was 30.00 (IQR 10.00-120.00) minutes. Each 30-minute increase in total daily and presleep screen time was associated with higher odds of insomnia (adjusted odds ratio [aOR] 1.14, 95% CI 1.05-1.23, and aOR 1.41, 95% CI 1.26-1.57, respectively). High exposure was associated with 2.46-fold (more than the median [334 minutes per day]) and 3.22-fold (more than the median [30 minutes]) higher odds of insomnia. Stratified by perceived stigma, participants with both high daily screen time and high stigma had the highest odds of insomnia (aOR 4.53, 95% CI 1.42-14.49). A similar pattern was observed for high presleep screen time and high stigma (aOR 2.59, 95% CI 1.54-4.37). However, effect measure modification was not significant on either the multiplicative or additive scale.

**Conclusions:**

This cross-sectional study revealed associations between screen time exposure and insomnia in patients with breast cancer, with more consistent findings for presleep screen time. Findings for total daily screen time should be interpreted cautiously because of extensive missing objective screen time data. These findings support further longitudinal studies and suggest that reducing presleep screen exposure may be considered in supportive oncology care.

## Introduction

Insomnia ranks among the most prevalent complications across cancer trajectories, manifesting as difficulties with sleep onset, maintenance, duration, and early-morning awakening [1]. Breast cancer demonstrates the strongest insomnia association among all types of malignancies, with epidemiological prevalence ranging from 42% to 69% [2]. In affected patients, insomnia is associated with cascading effects that diminish quality of life, worsen psychiatric symptoms, impair cognition, and contribute to adverse prognoses through bidirectional physical and psychological decline [3].

Prolonged screen exposure has emerged as an important public health concern, with particularly notable effects on sleep. Investigations of presleep screen use demonstrate that short-wavelength blue light emission suppresses melatonin secretion while concomitantly reducing delta waves (slow-wave sleep) and enhancing alpha waves (wakefulness associated), thereby disrupting sleep architecture [4,5]. Growing evidence links cumulative daily screen exposure to insomnia and related sleep problems, with interactive media (eg, smartphones) exerting stronger sleep-disruptive effects than passive media (eg, television) [6]. Smartphones have become ubiquitous owing to their portability, versatility, and interactive features, exerting increasing influence on daily life; however, few studies have objectively assessed screen time using device-based measures [7,8].

Perceived stigma may also be relevant to screen-related sleep disturbance in patients with breast cancer. Stigma’s relationship to breast cancer may involve internalized shame, perceived social rejection, altered body image (eg, alopecia and mastectomy), and reluctance to disclose illness-related concerns [9,10]. These experiences may increase psychological distress and promote smartphone use for information seeking, emotional support, or social connection. Empirically, stigma has been associated with poorer sleep quality among patients with breast cancer [11]. Therefore, we examined perceived stigma as a potential vulnerability factor that may modify the association between screen time and insomnia.

This study aimed to explore the association between smartphone screen time and insomnia in patients with breast cancer while examining effect modification by perceived stigma. We hypothesized that greater total and presleep screen time would be associated with higher odds of insomnia and that these associations may be stronger among patients reporting higher stigma as stigma heightens vulnerability and reliance on interactive devices. Understanding the association between screen time and insomnia is essential for developing targeted behavioral interventions that mitigate sleep disruption and improve quality of life during breast cancer treatment.

## Methods

### Data Sources and Population

Patients were recruited from the outpatient clinic of the Department of Breast Surgery, Longhua Hospital, Shanghai University of Traditional Chinese Medicine, from August 1, 2023, to February 20, 2024. Eligible participants were women aged 18 to 75 years with breast cancer diagnosed according to the National Comprehensive Cancer Network Clinical Practice Guidelines in Oncology (version 2.2022). Participants completed questionnaires on sleep patterns, screen use, and demographic characteristics. We excluded patients with preexisting insomnia that had begun before breast cancer diagnosis and remained ongoing at study enrollment. Of the initial cohort of 778 patients with breast cancer, 66 (8.5%) were excluded on this basis, leaving 712 (91.5%) eligible participants. Due to differential availability of screen time measurements, the analytic samples comprised 240 participants with complete data for the total daily screen time analysis and 644 participants with complete data for the presleep screen time analysis. The reporting of this study adhered to the STROBE (Strengthening the Reporting of Observational Studies in Epidemiology) guidelines for cross-sectional studies [12].

### Measurements

#### Exposure

Total daily screen time was objectively measured using built-in tracking apps on smartphones (Android, Google LLC; iOS, Apple Inc). These system modules automatically tracked average daily screen time in minutes over a 7-day rolling window. Prior mobile sensing research has demonstrated the feasibility of collecting passive smartphone use metrics, including screen-related behaviors, across iOS and Android platforms [13]. The secondary exposure was presleep screen time, assessed via structured questionnaires asking participants to report their typical phone use duration before sleep in minutes over the previous week. Both exposures were analyzed as continuous variables per 30-minute increment and as binary variables dichotomized at the median (334 minutes per day for total daily screen time and 30 minutes for presleep screen time) to facilitate interpretation and effect measure modification analyses.

#### Outcome

The primary outcome was insomnia (yes or no), diagnosed by licensed clinicians using a standardized clinical assessment based on the *Diagnostic and Statistical Manual of Mental Disorders, Fifth Edition*, criteria [14].

#### Covariates

On the basis of a directed acyclic graph (Figure S1 in Multimedia Appendix 1), we selected covariates assumed to be associated with both screen time and insomnia [1,7]. The adjustment set included demographic, cancer-related, and behavioral or physiological factors, including age; educational level; profession; time since breast cancer diagnosis; tumor, node, metastasis stage; current treatment status; menopause status; regular alcohol consumption; and daily working hours. Perceived stigma was assessed using the validated Social Impact Scale, which measures social exclusion, financial insecurity, inner shame, and social isolation, and was examined as a hypothesized effect modifier [15]. Total Social Impact Scale scores were dichotomized at the median to examine effect measure modification.

### Statistical Analysis

Complete-case analysis was used for the primary analysis, with patterns of missingness assessed by comparing characteristics between participants with and without missing values (Tables S1 and S2 in Multimedia Appendix 1). We used multivariable logistic regression to estimate odds ratios (ORs) and 95% CIs for associations between screen time exposures and insomnia using hierarchical adjustment across 3 models. Model 1 was adjusted for age, educational level, and profession. Model 2 was additionally adjusted for time since breast cancer diagnosis; tumor, node, metastasis stage; and current treatment. Model 3 was further adjusted for menopause status, regular alcohol consumption, and daily working hours. Effect measure modification by perceived stigma was assessed on both multiplicative and additive scales. Multiplicative interaction was evaluated using likelihood ratio tests, whereas additive interaction was quantified using the relative excess risk due to interaction (RERI), with 95% CIs calculated via the delta method.

### Sensitivity Analyses

We conducted several sensitivity analyses: (1) inverse probability of treatment weighting as an alternative method to control confounding; (2) g-computation implementing the parametric g-formula to estimate marginal effects under hypothetical screen time interventions; (3) imputation for missing data using median imputation (continuous variables) and mode imputation (categorical variables) to examine the effect of missing values when examining presleep screen time as exposure; (4) ordinal logistic regression using the Insomnia Severity Index (ISI) as an outcome, categorized as no clinically significant insomnia (0-7), subthreshold insomnia (8-14), moderate insomnia (15-21), and severe insomnia (22-28); and (5) ordinal logistic regression using the Pittsburgh Sleep Quality Index (PSQI) as an outcome, categorized as very good sleep (0-5), good sleep (6-10), poor sleep (11-15), and very poor sleep (16-21). For effect measure modification analyses in the ISI and PSQI sensitivity analyses, outcomes were dichotomized (ISI score≥8 indicating presence of insomnia symptoms; PSQI score>5 indicating poor sleep quality) to facilitate calculation of stratum-specific ORs and interaction measures. In addition, to assess robustness to unmeasured confounding, we calculated e-values for the binary screen time associations (high vs low) in the fully adjusted model [16]. Because insomnia was a common outcome, we approximated the risk ratio as the square root of the OR before computing the e-value, defined as the minimum strength of association on the risk ratio scale, that an unmeasured confounder would need to have with both the exposure and the outcome (beyond the measured covariates) to fully explain away the observed association. E-values were computed for both the point estimate and the CI limit closest to the null. All analyses were performed using the R statistical software (version 4.3.0 or later; R Foundation for Statistical Computing), with 2-sided tests and significance set at a *P* value below .05.

### Ethical Considerations

This study was conducted in accordance with the Declaration of Helsinki and was approved by the ethics committee of Longhua Hospital, Shanghai University of Traditional Chinese Medicine (approval 2023LCSY045). All participants provided written informed consent prior to taking part in the study.

## Results

**Figure 1.**
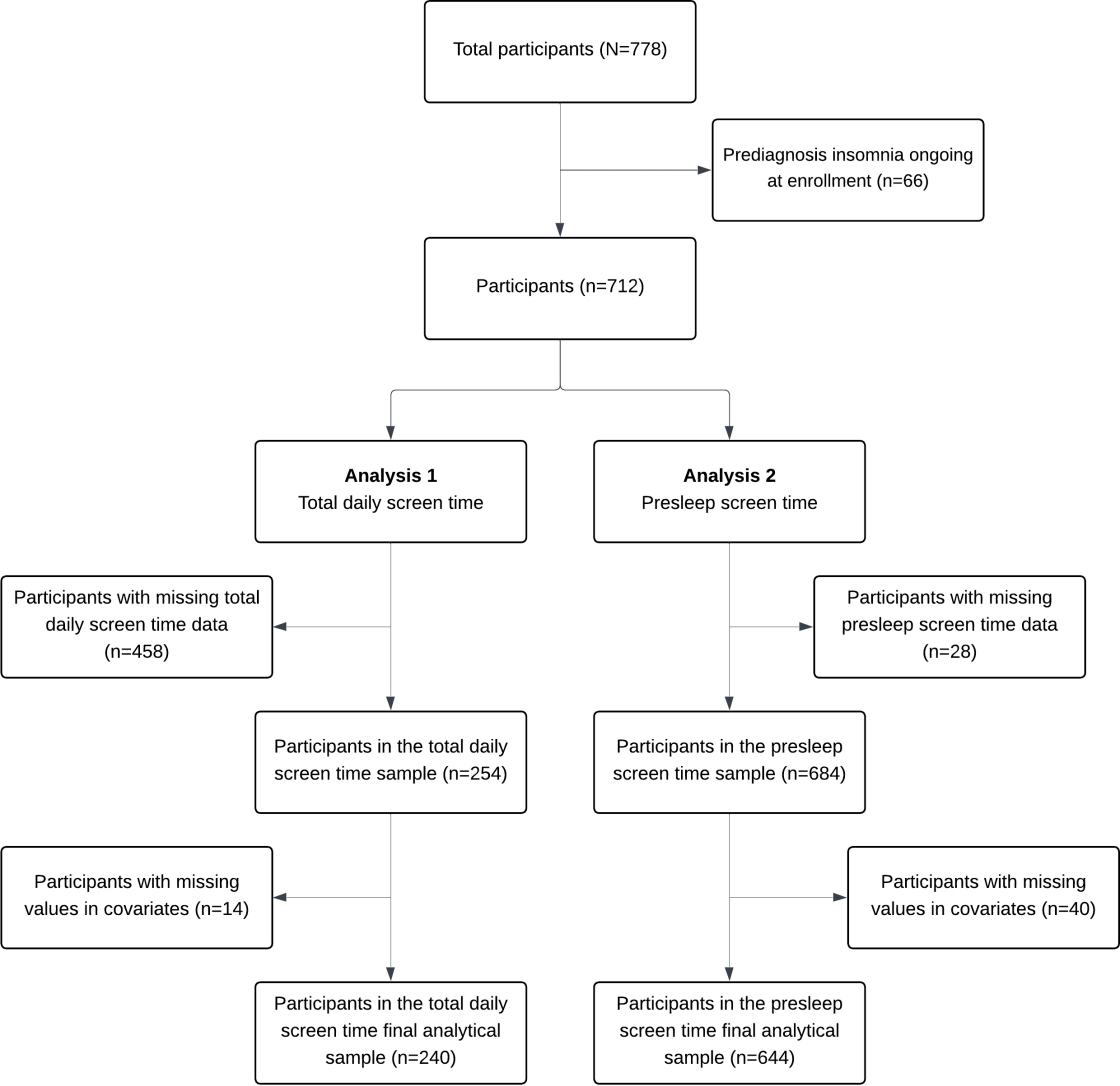
Flowchart of participants.

This study aimed to examine the association between screen time and insomnia among patients with breast cancer and effect measure modification by perceived stigma. The analytic samples comprised 240 participants in the total daily screen time analysis and 644 participants in the presleep screen time analysis (Figure 1). In the total daily screen time sample, mean age was 51.82 (SD 10.62) years, median time since diagnosis was 14.00 (IQR 7.00-29.25) months, and 77.5% (186/240) had an insomnia diagnosis. In the presleep screen time sample, mean age was 53.46 (SD 10.76) years, median time since diagnosis was 15.00 (IQR 7.00-36.00) months, and 65.1% (419/644) had an insomnia diagnosis (Table 1).

The median total daily screen time was 334.00 (IQR 232.50-440.25) minutes, and median presleep screen time was 30.00 (IQR 10.00-120.00) minutes. Participants with high total daily screen time were younger (mean age 48.94, SD 9.94 vs 54.74, SD 10.54 years; *P*<.001) and more likely to be engaged in mental work (77/121, 63.6% vs 33/119, 27.7%; *P*<.001). Those with high presleep screen time had higher rates of insomnia diagnosis (247/319, 77.4% vs 172/325, 52.9%; *P*<.001; Table 1).

**Figure 2.**
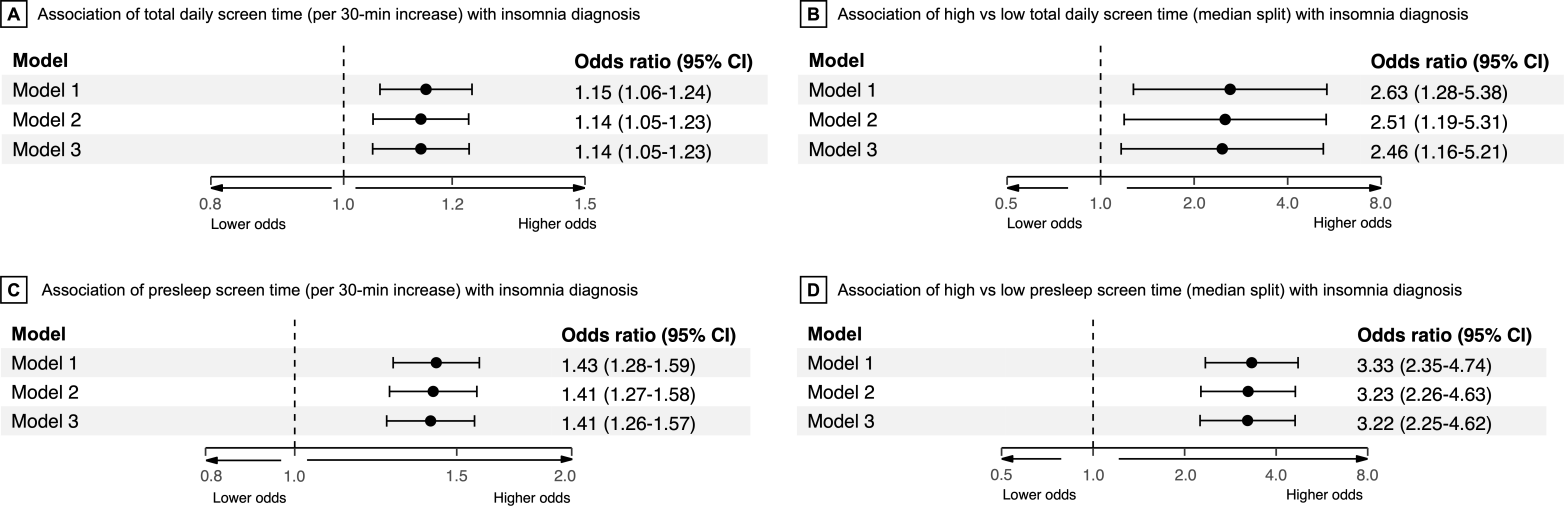
Association between both total daily screen time and presleep screen time and insomnia.

In fully adjusted models, each 30-minute increase in total daily screen time was associated with 14% increased odds of insomnia (adjusted OR [aOR] 1.14, 95% CI 1.05-1.23), whereas each 30-minute increase in presleep screen time was associated with 41% increased odds of insomnia (aOR 1.41, 95% CI 1.26-1.57). When analyzed as binary variables, high total daily screen time (more than the median [334 minutes per day]) was associated with 2.46-fold increased odds of insomnia (95% CI 1.16-5.21), and high presleep screen time (more than the median [30 minutes]) was associated with 3.22-fold increased odds of insomnia (95% CI 2.25-4.62) compared with low–screen time groups (Figure 2).

**Table 1.** Participant characteristics.

	Total daily screen time sample (n=240)					Presleep screen time sample (n=644)				
	Overall	Low (n=119)	High (n=121)	*P* value	Standardized mean difference	Overall	Low (n=325)	High (n=319)	*P* value	Standardized mean difference
Age (y), mean (SD)	51.82 (10.62)	54.74 (10.54)	48.94 (9.94)	<.001	0.566	53.46 (10.76)	54.07 (10.64)	52.83 (10.86)	.14	0.115
Educational level, n (%)				<.001	0.844				.85	0.045
Lower than high school	121 (50.4)	37 (31.1)	84 (69.4)			281 (43.6)	141 (43.4)	140 (43.9)		
High school	65 (27.1)	42 (35.3)	23 (19)			163 (25.3)	80 (24.6)	83 (26)		
Higher than high school	54 (22.5)	40 (33.6)	14 (11.6)			200 (31.1)	104 (32)	96 (30.1)		
Profession, n (%)				<.001	0.796				.90	0.061
Manual work	7 (2.9)	3 (2.5)	4 (3.3)			25 (3.9)	11 (3.4)	14 (4.4)		
Retired	106 (44.2)	72 (60.5)	34 (28.1)			294 (45.7)	147 (45.2)	147 (46.1)		
Mental work	110 (45.8)	33 (27.7)	77 (63.6)			281 (43.6)	145 (44.6)	136 (42.6)		
Not retired but not working	17 (7.1)	11 (9.2)	6 (5)			44 (6.8)	22 (6.8)	22 (6.9)		
Time since breast cancer diagnosis (mo), median (IQR)	14.00 (7.00-29.25)	14.00 (7.00-29.50)	14.00 (8.00-28.00)	.88	0.087	15.00 (7.00-36.00)	18.00 (7.00-33.00)	14.00 (7.00-36.00)	.90	0.076
TNM^a^, n (%)				.07	0.383				.15	0.207
Stage 0	1 (0.4)	1 (0.8)	0 (0)			12 (1.9)	10 (3.1)	2 (0.6)		
Stage 1	83 (34.6)	40 (33.6)	43 (35.5)			239 (37.1)	119 (36.6)	120 (37.6)		
Stage 2	90 (37.5)	38 (31.9)	52 (43)			244 (37.9)	127 (39.1)	117 (36.7)		
Stage 3	52 (21.7)	29 (24.4)	23 (19)			111 (17.2)	50 (15.4)	61 (19.1)		
Stage 4	14 (5.8)	11 (9.2)	3 (2.5)			38 (5.9)	19 (5.8)	19 (6)		
Current breast cancer treatment, n (%)	225 (93.8)	111 (93.3)	114 (94.2)	.80	0.039	600 (93.2)	298 (91.7)	302 (94.7)	.16	0.118
Menopause, n (%)	185 (77.1)	99 (83.2)	86 (71.1)	.03	0.292	514 (79.8)	259 (79.7)	255 (79.9)	≥.99	0.006
Regular alcohol consumption, n (%)	2 (0.8)	1 (0.8)	1 (0.8)	≥.99	0.002	11 (1.7)	7 (2.2)	4 (1.3)	.55	0.07
Daily working hours, n (%)				<.001	0.633				.70	0.066
Not working or studying	103 (42.9)	69 (58.0)	34 (28.1)			278 (43.2)	137 (42.2)	141 (44.2)		
<8 h per d	102 (42.5)	37 (31.1)	65 (53.7)			281 (43.6)	147 (45.2)	134 (42)		
≥8 h per d	35 (14.6)	13 (10.9)	22 (18.2)			85 (13.2)	41 (12.6)	44 (13.8)		
Total daily screen time or presleep screen time (min), median (IQR)	334.00 (232.50-440.25)	231.00 (158.00-282.50)	440.00 (387.00-516.00)	<.001	2.71	30.00 (10.00-120.00)	10.00 (0.00-30.00)	120.00 (60.00-120.00)	<.001	2.256
SIS^b^ (range 24-96)										
Social exclusion, median (IQR)	14.00 (11.00-19.00)	15.00 (10.50-18.00)	14.00 (11.00-19.00)	.81	0.015	16.00 (11.00-19.00)	16.00 (10.00-18.00)	16.00 (11.00-19.00)	.10	0.127
Financial insecurity, median (IQR)	6.00 (3.00-6.00)	6.00 (3.00-6.00)	6.00 (3.00-6.00)	.77	0.047	6.00 (3.00-6.00)	6.00 (3.00-6.00)	6.00 (3.00-7.00)	.08	0.136
Inner shame, median (IQR)	10.00 (5.00-11.00)	10.00 (5.00-11.00)	10.00 (6.00-11.00)	.63	0.035	10.00 (5.00-11.00)	10.00 (5.00-11.00)	10.00 (5.00-11.00)	.16	0.112
Social isolation, median (IQR)	12.00 (7.00-14.00)	12.00 (7.00-14.00)	12.00 (7.00-14.00)	.74	0.019	14.00 (7.00-15.00)	14.00 (7.00-14.00)	13.00 (7.00-15.00)	.16	0.107
Total score, median (IQR)	42.00 (29.00-51.00)	43.00 (27.00-50.00)	41.00 (30.00-51.00)	.67	0.027	46.00 (29.00-51.00)	46.00 (29.00-50.00)	45.00 (30.00-52.00)	.09	0.129
High SIS score, n (%)	105 (43.8)	52 (43.7)	53 (43.8)	≥.99	0.002	323 (50.2)	164 (50.5)	159 (49.8)	.94	0.012
Insomnia diagnosis, n (%)	186 (77.5)	86 (72.3)	100 (82.6)	.06	0.25	419 (65.1)	172 (52.9)	247 (77.4)	<.001	0.532
ISI^c^										
Total score (0-28), median (IQR)	7.00 (4.00-11.00)	7.00 (3.00-11.00)	6.00 (4.00-11.00)	.79	0.078	6.00 (3.00-10.25)	4.00 (2.00-9.00)	7.00 (4.00-11.00)	<.001	0.379
Category, n (%)				.35	0.235				.003	0.301
No clinically significant insomnia	133 (55.4)	65 (54.6)	68 (56.2)			388 (60.2)	218 (67.1)	170 (53.3)		
Subthreshold insomnia	80 (33.3)	42 (35.3)	38 (31.4)			169 (26.2)	72 (22.2)	97 (30.4)		
Moderate insomnia	24 (10)	12 (10.1)	12 (9.9)			72 (11.2)	31 (9.5)	41 (12.9)		
Severe insomnia	3 (1.3)	0 (0)	3 (2.5)			15 (2.3)	4 (1.2)	11 (3.4)		
PSQI^d^										
Total score (0-21), median (IQR)	8.00 (5.00-11.00)	8.00 (5.00-11.00)	8.00 (5.00-11.00)	.89	0.046	7.00 (4.00-11.00)	6.00 (3.00-9.00)	9.00 (6.00-12.00)	<.001	0.56
Category, n (%)				.54	0.19				<.001	0.514
Very good sleep	68 (28.3)	33 (27.7)	35 (28.9)			231 (35.9)	154 (47.4)	77 (24.1)		
Good sleep	100 (41.7)	51 (42.9)	49 (40.5)			244 (37.9)	108 (33.2)	136 (42.6)		
Poor sleep	64 (26.7)	33 (27.7)	31 (25.6)			142 (22)	52 (16)	90 (28.2)		
Very poor sleep	8 (3.3)	2 (1.7)	6 (5)			27 (4.2)	11 (3.4)	16 (5)		

a TNM: tumor, node, metastasis.

b SIS: Social Impact Scale.

c ISI: Insomnia Severity Index.

d PSQI: Pittsburgh Sleep Quality Index.

Perceived stigma did not significantly modify the association between screen time and insomnia. Among participants with low perceived stigma, high total daily screen time was not significantly associated with insomnia (aOR 1.69, 95% CI 0.67-4.30), whereas among those with high perceived stigma, high total daily screen time was associated with 4.5 times higher odds of insomnia (aOR 4.53, 95% CI 1.42-14.49). However, tests for effect modification were not statistically significant on either the multiplicative scale (ratio of ORs=2.68, 95% CI 0.65-11.11) or the additive scale (RERI=3.07, 95% CI –1.77 to 7.91; Table 2). For presleep screen time, associations with insomnia were strong in both the low-stigma (aOR 4.01, 95% CI 2.44-6.60) and high-stigma (aOR 2.59, 95% CI 1.54-4.37) groups, with no evidence of effect modification on either scale (RERI=0.14, 95% CI –2.52 to 2.80; ratio of ORs=0.65, 95% CI 0.32-1.32; Table 3). Although the stratum-specific estimates suggested a stronger association between high total daily screen time and insomnia among participants with high perceived stigma, the CIs were wide, and formal tests for interaction were not statistically significant. These findings should therefore be interpreted as exploratory.

**Table 2.** Effect measure modification by perceived stigma on the association between total daily screen time and insomnia diagnosis.

	Low screen time			High screen time			Insomnia with strata of perceived stigma, OR^a^ (95% CI)
	Participants with insomnia, n	Participants without insomnia, n	OR (95% CI)	Participants with insomnia, n	Participants without insomnia, n	OR (95% CI)	
Low perceived stigma^b^^c^	49	18	1.0 (reference)	53	15	1.69 (0.67-4.3)	1.69 (0.67-4.3)
High perceived stigma	37	15	1.07 (0.43-2.61)	47	6	4.83 (1.54-15.12)	4.53 (1.42-14.49)

a OR: odds ratio. ORs are adjusted for age; educational level; profession; time since breast cancer diagnosis; tumor, node, metastasis stage; current treatment; menopause status; regular alcohol consumption; and daily working hours.

b Measure of effect measure modification on the additive scale: relative excess risk due to interaction=3.07 (95% CI –1.77 to 7.91).

c Measure of effect measure modification on the multiplicative scale: ratio of odds ratios=2.68 (95% CI 0.65‐11.11).

Sensitivity analyses yielded generally consistent results with some attenuation. Using inverse probability of treatment weighting, associations remained significant for both high total daily screen time (aOR 2.61, 95% CI 1.19-5.72) and high presleep screen time (aOR 2.86, 95% CI 1.99-4.09; Figure S2 in Multimedia Appendix 1). With g-computation, the association for high total daily screen time was no longer statistically significant (aOR 2.17, 95% CI 0.99-4.79), whereas high presleep screen time maintained a strong association (aOR 3.00, 95% CI 2.16-4.17; Figure S3 in Multimedia Appendix 1). When missing presleep screen time data were handled using median or mode imputation, the association between high presleep screen time and insomnia remained robust (aOR 2.55, 95% CI 1.83-3.57; Figure S4 in Multimedia Appendix 1). Using ordinal logistic regression with ISI and PSQI scores as outcomes, high presleep screen time remained significantly associated with worse outcomes (ISI: aOR 1.87, 95% CI 1.35-2.59; PSQI: aOR 2.52, 95% CI 1.87-3.40), whereas high total daily screen time showed nonsignificant associations (ISI: aOR 1.28, 95% CI 0.70-2.34; PSQI: aOR 1.54, 95% CI 0.88-2.70; Figures S5 and S6 in Multimedia Appendix 1). Effect modification findings remained exploratory across sensitivity analyses (Tables S3-S11 in Multimedia Appendix 1). E-value analysis showed that an unmeasured confounder would need to be associated with both high presleep screen time and insomnia by a risk ratio of at least 2.99 (2.37 for the lower confidence limit) to fully account for the observed association. The corresponding e-values for high total daily screen time were more modest (2.51 for the point estimate; 1.37 for the lower confidence limit), in accordance with the less consistent findings for this exposure across sensitivity analyses.

**Table 3.** Effect measure modification by perceived stigma on the association between presleep screen time and insomnia diagnosis.

	Low screen time			High screen time			Insomnia with strata of perceived stigma, OR^a^ (95% CI)
	Participants with insomnia, n	Participants without insomnia, n	OR (95% CI)	Participants with insomnia, n	Participants without insomnia, n	OR (95% CI)	
Low perceived stigma^b^^c^	71	90	1.0 (reference)	120	40	4.01 (2.44-6.6)	4.01 (2.44-6.6)
High perceived stigma	101	63	1.98 (1.25-3.15)	127	32	5.14 (3.05-8.64)	2.59 (1.54-4.37)

a OR: odds ratio. ORs are adjusted for age; educational level; profession; time since breast cancer diagnosis; tumor, node, metastasis stage; current treatment; menopause status; regular alcohol consumption; and daily working hours.

b Measure of effect measure modification on the additive scale: relative excess risk due to interaction=0.14 (95% CI –2.52 to 2.8).

c Measure of effect measure modification on the multiplicative scale: ratio of odds ratios=0.65 (95% CI 0.32‐1.32).

## Discussion

This cross-sectional analysis found that both total daily and presleep screen time were associated with higher odds of insomnia among patients with breast cancer, with a stronger association for presleep screen time. However, evidence on total daily screen time was less consistent in sensitivity analyses and should be interpreted with considerable caution because of extensive missing objective screen time data and possible selection bias. Perceived stigma did not significantly modify either association.

Our findings align with literature associating digital media exposure with adverse sleep outcomes, including inadequate sleep duration, poor quality, daytime somnolence, and various types of insomnia problems, primarily documented in school-aged cohorts [7,17]. Although most previous studies have focused on adolescents and young adults, our results suggest that screen exposure may also be relevant to sleep health in patients with breast cancer, a population with a high burden of insomnia. Given the clinical burden of insomnia in oncology, screen-related sleep problems warrant further investigation in breast cancer supportive care.

Several mechanisms may explain the association between presleep screen exposure and insomnia. Evening exposure to light-emitting screens may delay circadian timing, suppress melatonin secretion, and increase cognitive or emotional arousal before bedtime [4,5,18]. In contrast, the role of perceived stigma is more speculative. Perceived stigma is clinically relevant to sleep health in patients with breast cancer and may reflect a vulnerability context for screen-related sleep disturbance [9-11,19]. However, the effect modification analyses did not provide statistically significant evidence that perceived stigma modified the associations between screen exposure and insomnia on either the multiplicative or additive scale. Therefore, the observed stratum-specific patterns should be interpreted as exploratory and hypothesis generating rather than confirmatory and require confirmation in larger longitudinal studies.

This study has several limitations. First, the cross-sectional design precluded determination of temporality, and reverse causation remains possible because patients with insomnia may use smartphones when they have difficulty falling asleep or during nocturnal awakenings. To mitigate this concern, we excluded participants with insomnia that began before breast cancer diagnosis and remained ongoing at enrollment; however, reverse causation remains possible. Second, exposure assessment differed between the 2 screen time indicators. Measurement error in smartphone-recorded total daily screen time may have been nondifferential by insomnia status, but the direction and magnitude of bias cannot be predicted with certainty in multivariable analyses involving categorized exposures and common outcomes. Self-reported presleep screen time may be differentially misclassified. Future studies should use objective time-stamped measures of presleep exposure and consider probabilistic bias analysis. Third, incomplete screen time data, particularly for total daily screen time, may have introduced selection bias. Comparisons of participants with and without complete screen time data can be found in Tables S1 and S2 in Multimedia Appendix 1 to evaluate missingness patterns. Participants with complete total daily screen time data had a higher prevalence of insomnia than those with missing data, suggesting possible selection bias; therefore, findings for total daily screen time should be interpreted with considerable caution. Fourth, because insomnia was common, especially in the total daily screen time sample, ORs may overestimate relative risks and should be interpreted accordingly. Finally, we did not assess the content, purpose, or emotional engagement of smartphone use. Several psychological factors that may influence both screen use and sleep, such as anxiety, depression, and psychiatric history, were not measured and could contribute to residual confounding. Although e-value analysis quantified the strength of unmeasured confounding that would be needed to explain away the observed associations, it does not eliminate the possibility of residual confounding by these unmeasured psychological factors.

In conclusion, greater presleep smartphone screen time was consistently associated with higher odds of insomnia among patients with breast cancer, whereas findings for total daily screen time were less consistent and should be interpreted cautiously because of extensive missing objective screen time data. Perceived stigma did not significantly modify these associations. These findings support larger longitudinal studies using objective screen exposure measures and more detailed assessment of screen use context.

## Supplementary material

10.2196/85837 Multimedia Appendix 1 Supplementary material.

10.2196/85837 Checklist 1 STROBE checklist.

## Abbreviations

aOR: adjusted odds ratio
ISI: Insomnia Severity Index
OR: odds ratio
PSQI: Pittsburgh Sleep Quality Index
RERI: relative excess risk due to interaction
STROBE: Strengthening the Reporting of Observational Studies in Epidemiology
