# Supplementary material for: Association Between Screen Exposure and Insomnia Among Patients With Breast Cancer: Cross-Sectional Study
Source: J Med Internet Res. 2026 Jul 23;28:e85837. doi: 10.2196/85837 (PMC13394888; doi:10.2196/85837)
Supplement: Multimedia Appendix 1 [file jmir-v28-e85837-s001.docx]

**Supplementary Material**

**[eFigure 1](#_Toc202971164)** [Directed Acyclic Graph 3](#_Toc202971164)

**[eTable 1](#_Toc202971165)** [Comparison of Characteristics between Participants with and without Missing Values in the Daily Screen Time Sample 4](#_Toc202971165)

**[eTable 2](#_Toc202971166)** [Comparison of Characteristics between Participants with and without Missing Values in the Presleep Screen Time Sample 5](#_Toc202971166)

**[eFigure 2](#_Toc202971167)** [Sensitivity Analysis for Association Between Total Daily Screen Time, Presleep Screen Time, and Insomnia Disorder with Inverse probability Treatment Weighting 6](#_Toc202971167)

**[eTable 3](#_Toc202971168)** [Sensitivity Analysis for Effect Measure Modification by Perceived Stigma on the Association between Total Daily Screen Time and Insomnia Disorder with Inverse probability Treatment Weighting 7](#_Toc202971168)

**[eTable 4](#_Toc202971169)** [Sensitivity Analysis for Effect Measure Modification by Perceived Stigma on the Association between Presleep Screen Time and Insomnia Disorder with Inverse probability Treatment Weighting 8](#_Toc202971169)

**[eFigure 3](#_Toc202971170)** [Sensitivity Analysis for Association Between Total Daily Screen Time, Presleep Screen Time, and Insomnia Disorder with G-computation 9](#_Toc202971170)

**[eTable 5](#_Toc202971171)** [Sensitivity Analysis for Effect Measure Modification by Perceived Stigma on the Association between Total Daily Screen Time and Insomnia Disorder with G-computation 10](#_Toc202971171)

**[eTable 6](#_Toc202971172)** [Sensitivity Analysis for Effect Measure Modification by Perceived Stigma on the Association between Presleep Screen Time and Insomnia Disorder with G-computation 11](#_Toc202971172)

**[eFigure 4](#_Toc202971173)** [Sensitivity Analysis for Association Between Total Daily Screen Time, Presleep Screen Time, and Insomnia Disorder with Median/Mode Imputation](#_Toc202971173) ^[a](#_Toc202971173)^ [12](#_Toc202971173)

**[eTable 7](#_Toc202971174)** [Sensitivity Analysis for Effect Measure Modification by Perceived Stigma on the Association between Presleep Screen Time and Insomnia Disorder with Median/Mode Imputation](#_Toc202971174) ^[a](#_Toc202971174)^ [13](#_Toc202971174)

**[eFigure 5](#_Toc202971175)** [Sensitivity Analysis for Association Between Total Daily Screen Time, Presleep Screen Time, and Insomnia Severity 14](#_Toc202971175)

**[eTable 8](#_Toc202971176)** [Sensitivity Analysis for Effect Measure Modification by Perceived Stigma on the Association between Total Daily Screen Time and Insomnia Severity 15](#_Toc202971176)

**[eTable 9](#_Toc202971177)** [Sensitivity Analysis for Effect Measure Modification by Perceived Stigma on the Association between Presleep Screen Time and Insomnia Severity 16](#_Toc202971177)

**[eFigure 6](#_Toc202971178)** [Sensitivity Analysis for Association Between Total Daily Screen Time, Presleep Screen Time, and Sleep Quality 17](#_Toc202971178)

**[eTable 10](#_Toc202971179)** [Sensitivity Analysis for Effect Measure Modification by Perceived Stigma on the Association between Total Daily Screen Time and Sleep Quality 18](#_Toc202971179)

**[eTable 11](#_Toc202971180)** [Sensitivity Analysis for Effect Measure Modification by Perceived Stigma on the Association between Presleep Screen Time and Sleep Quality 19](#_Toc202971180)

# **eFigure 1** Directed Acyclic Graph

**
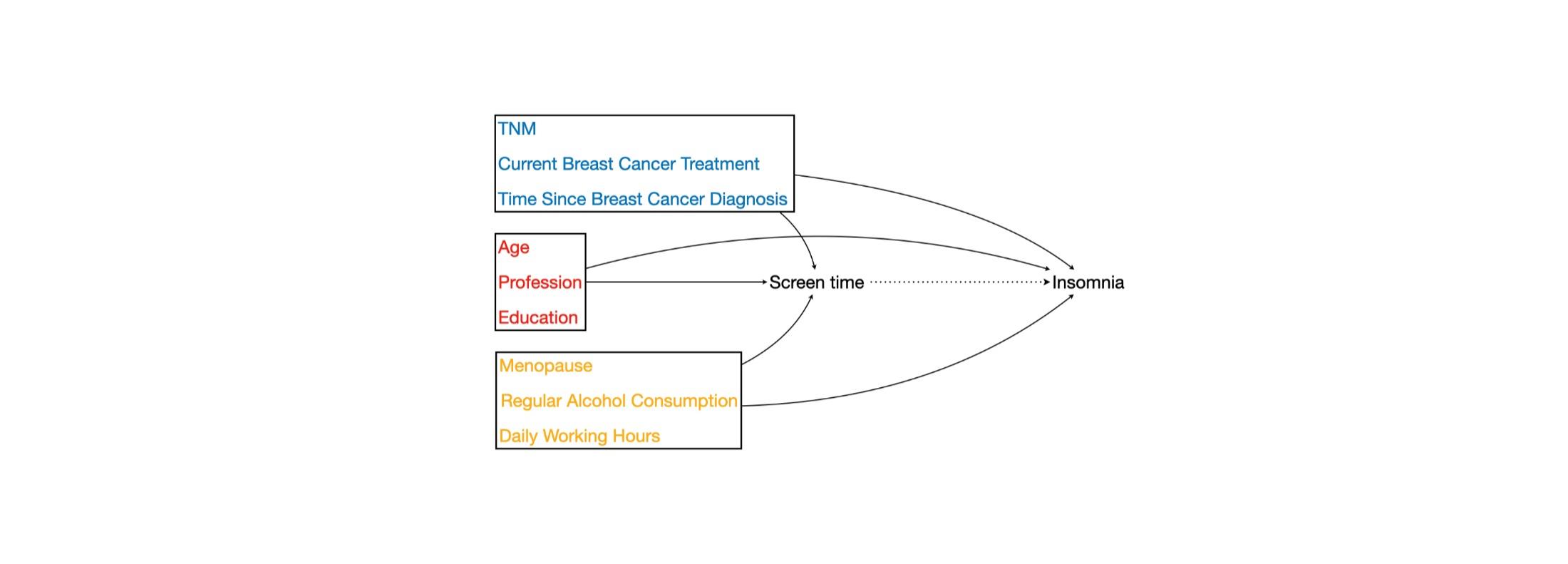
**

# **eTable 1** Comparison of Characteristics between Participants with and without Missing Values in the Total Daily Screen Time Analysis

|  | **Overall** | **Without Missing Value** | **With Missing Value** | ***p*** | **Standardized Mean Difference** | **Missing %** |
| --- | --- | --- | --- | --- | --- | --- |
| n | 712 | 240 | 472 |  |  |  |
| **Age, mean (SD), year** | 53.38 (10.82) | 51.82 (10.62) | 54.17 (10.84) | 0.006 | 0.219 | 0 |
| **Education, n (%)** |  |  |  | 0.002 | 0.293 | 0 |
| Below High School | 313 (44.0) | 121 (50.4) | 192 (40.7) |  |  |  |
| High School | 177 (24.9) | 65 (27.1) | 112 (23.7) |  |  |  |
| Above High School | 222 (31.2) | 54 (22.5) | 168 (35.6) |  |  |  |
| **Profession, n (%)** |  |  |  | 0.531 | 0.12 | 0.3 |
| Manual Work | 29 (4.1) | 7 (2.9) | 22 (4.7) |  |  |  |
| Retired | 327 (46.1) | 106 (44.2) | 221 (47.0) |  |  |  |
| Mental Work | 305 (43.0) | 110 (45.8) | 195 (41.5) |  |  |  |
| Not Retired but Not Working | 49 (6.9) | 17 (7.1) | 32 (6.8) |  |  |  |
| **Time Since Breast Cancer Diagnosis, median [IQR], month** | 17.00 [7.00, 36.00] | 14.00 [7.00, 29.25] | 19.00 [7.00, 37.00] | 0.043 | 0.138 | 0 |
| **TNM, n (%)** |  |  |  | 0.096 | 0.237 | 5.5 |
| Stage 0 | 12 (1.8) | 1 (0.4) | 11 (2.5) |  |  |  |
| Stage 1 | 249 (37.0) | 83 (34.6) | 166 (38.3) |  |  |  |
| Stage 2 | 255 (37.9) | 90 (37.5) | 165 (38.1) |  |  |  |
| Stage 3 | 119 (17.7) | 52 (21.7) | 67 (15.5) |  |  |  |
| Stage 4 | 38 (5.6) | 14 (5.8) | 24 (5.5) |  |  |  |
| **Current Breast Cancer Treatment, n (%)** | 665 (93.4) | 225 (93.8) | 440 (93.2) | 0.874 | 0.021 | 0 |
| **Menopause, n (%)** | 565 (79.4) | 185 (77.1) | 380 (80.5) | 0.283 | 0.084 | 0 |
| **Regular Alcohol Consumption, n (%)** | 12 (1.7) | 2 (0.8) | 10 (2.1) | 0.355 | 0.107 | 0 |
| **Daily Working Hours, n (%)** |  |  |  | 0.582 | 0.081 | 0 |
| Not Working/Studying | 315 (44.2) | 103 (42.9) | 212 (44.9) |  |  |  |
| <8 hours/day | 306 (43.0) | 102 (42.5) | 204 (43.2) |  |  |  |
| >= 8 hours/day | 91 (12.8) | 35 (14.6) | 56 (11.9) |  |  |  |
| **Total Daily Screen Time, median [IQR], minute** | 331.50 [241.75, 439.75] | 334.00 [232.50, 440.25] | 322.50 [273.00, 401.00] | 0.825 | 0.056 | 64.3 |
| **Social Impact Scale: Social Exclusion, median [IQR]** | 16.00 [11.00, 19.00] | 14.00 [11.00, 19.00] | 17.00 [11.00, 19.00] | 0.354 | 0.112 | 0.1 |
| **Social Impact Scale: Financial Insecurity, median [IQR]** | 6.00 [3.00, 6.00] | 6.00 [3.00, 6.00] | 6.00 [3.00, 6.00] | 0.767 | 0.029 | 0.1 |
| **Social Impact Scale: Inner Shame, median [IQR]** | 10.00 [5.00, 11.00] | 10.00 [5.00, 11.00] | 10.00 [5.00, 11.00] | 0.94 | 0.017 | 0.1 |
| **Social Impact Scale: Social Isolation, median [IQR]** | 14.00 [7.00, 15.00] | 12.00 [7.00, 14.00] | 14.00 [7.00, 15.00] | 0.141 | 0.126 | 0.1 |
| **Insomnia Disorder, n (%)** | 463 (65.0) | 186 (77.5) | 277 (58.7) | <0.001 | 0.412 | 0 |
| **Insomnia Severity Index (ISI), median [IQR]** | 6.00 [3.00, 11.00] | 7.00 [4.00, 11.00] | 6.00 [2.00, 10.00] | 0.023 | 0.095 | 0 |
| **Pittsburgh Sleep Quality Index (PSQI), median [IQR]** | 7.00 [4.00, 11.00] | 8.00 [5.00, 11.00] | 7.00 [4.00, 11.00] | 0.002 | 0.219 | 0 |

# **eTable 2** Comparison of Characteristics between Participants with and without Missing Values in the Presleep Screen Time Analysis

|  | **Overall** | **Without Missing Value** | **With Missing Value** | ***p*** | **Standardized Mean Difference** | **Missing %** |
| --- | --- | --- | --- | --- | --- | --- |
| n | 712 | 644 | 68 |  |  |  |
| **Age, mean (SD), year** | 53.38 (10.82) | 53.46 (10.76) | 52.60 (11.44) | 0.557 | 0.077 | 0 |
| **Education, n (%)** |  |  |  | 0.688 | 0.113 | 0 |
| Below High School | 313 (44.0) | 281 (43.6) | 32 (47.1) |  |  |  |
| High School | 177 (24.9) | 163 (25.3) | 14 (20.6) |  |  |  |
| Above High School | 222 (31.2) | 200 (31.1) | 22 (32.4) |  |  |  |
| **Profession, n (%)** |  |  |  | 0.631 | 0.166 | 0.3 |
| Manual Work | 29 (4.1) | 25 (3.9) | 4 (6.1) |  |  |  |
| Retired | 327 (46.1) | 294 (45.7) | 33 (50.0) |  |  |  |
| Mental Work | 305 (43.0) | 281 (43.6) | 24 (36.4) |  |  |  |
| Not Retired but Not Working | 49 (6.9) | 44 (6.8) | 5 (7.6) |  |  |  |
| **Time Since Breast Cancer Diagnosis, median [IQR], month** | 17.00 [7.00, 36.00] | 15.00 [7.00, 36.00] | 25.50 [9.00, 53.00] | 0.008 | 0.286 | 0 |
| **TNM, n (%)** |  |  |  | 0.408 | 0.465 | 5.5 |
| Stage 0 | 12 (1.8) | 12 (1.9) | 0 (0.0) |  |  |  |
| Stage 1 | 249 (37.0) | 239 (37.1) | 10 (34.5) |  |  |  |
| Stage 2 | 255 (37.9) | 244 (37.9) | 11 (37.9) |  |  |  |
| Stage 3 | 119 (17.7) | 111 (17.2) | 8 (27.6) |  |  |  |
| Stage 4 | 38 (5.6) | 38 (5.9) | 0 (0.0) |  |  |  |
| **Current Breast Cancer Treatment, n (%)** | 665 (93.4) | 600 (93.2) | 65 (95.6) | 0.61 | 0.105 | 0 |
| **Menopause, n (%)** | 565 (79.4) | 514 (79.8) | 51 (75.0) | 0.347 | 0.115 | 0 |
| **Regular Alcohol Consumption, n (%)** | 12 (1.7) | 11 (1.7) | 1 (1.5) | 1 | 0.019 | 0 |
| **Daily Working Hours, n (%)** |  |  |  | 0.187 | 0.236 | 0 |
| Not Working/Studying | 315 (44.2) | 278 (43.2) | 37 (54.4) |  |  |  |
| <8 hours/day | 306 (43.0) | 281 (43.6) | 25 (36.8) |  |  |  |
| >= 8 hours/day | 91 (12.8) | 85 (13.2) | 6 (8.8) |  |  |  |
| **Presleep Screen Time, median [IQR], minute** | 30.00 [10.00, 120.00] | 30.00 [10.00, 120.00] | 60.00 [0.00, 120.00] | 0.837 | 0.056 | 3.9 |
| **Social Impact Scale: Social Exclusion, median [IQR]** | 16.00 [11.00, 19.00] | 16.00 [11.00, 19.00] | 16.00 [9.50, 19.00] | 0.515 | 0.037 | 0.1 |
| **Social Impact Scale: Financial Insecurity, median [IQR]** | 6.00 [3.00, 6.00] | 6.00 [3.00, 6.00] | 5.00 [3.00, 6.50] | 0.504 | 0.082 | 0.1 |
| **Social Impact Scale: Inner Shame, median [IQR]** | 10.00 [5.00, 11.00] | 10.00 [5.00, 11.00] | 10.00 [5.00, 11.00] | 0.525 | 0.017 | 0.1 |
| **Social Impact Scale: Social Isolation, median [IQR]** | 14.00 [7.00, 15.00] | 14.00 [7.00, 15.00] | 12.00 [7.00, 15.00] | 0.383 | 0.092 | 0.1 |
| **Insomnia Disorder, n (%)** | 463 (65.0) | 419 (65.1) | 44 (64.7) | 1 | 0.007 | 0 |
| **Insomnia Severity Index (ISI), median [IQR]** | 6.00 [3.00, 11.00] | 6.00 [3.00, 10.25] | 7.00 [2.00, 11.00] | 0.686 | 0.026 | 0 |
| **Pittsburgh Sleep Quality Index (PSQI), median [IQR]** | 7.00 [4.00, 11.00] | 7.00 [4.00, 11.00] | 8.00 [4.00, 12.00] | 0.599 | 0.079 | 0 |

# **eFigure 2** Sensitivity analysis for association between total daily screen time, presleep screen time, and insomnia disorder with inverse probability treatment weighting

**
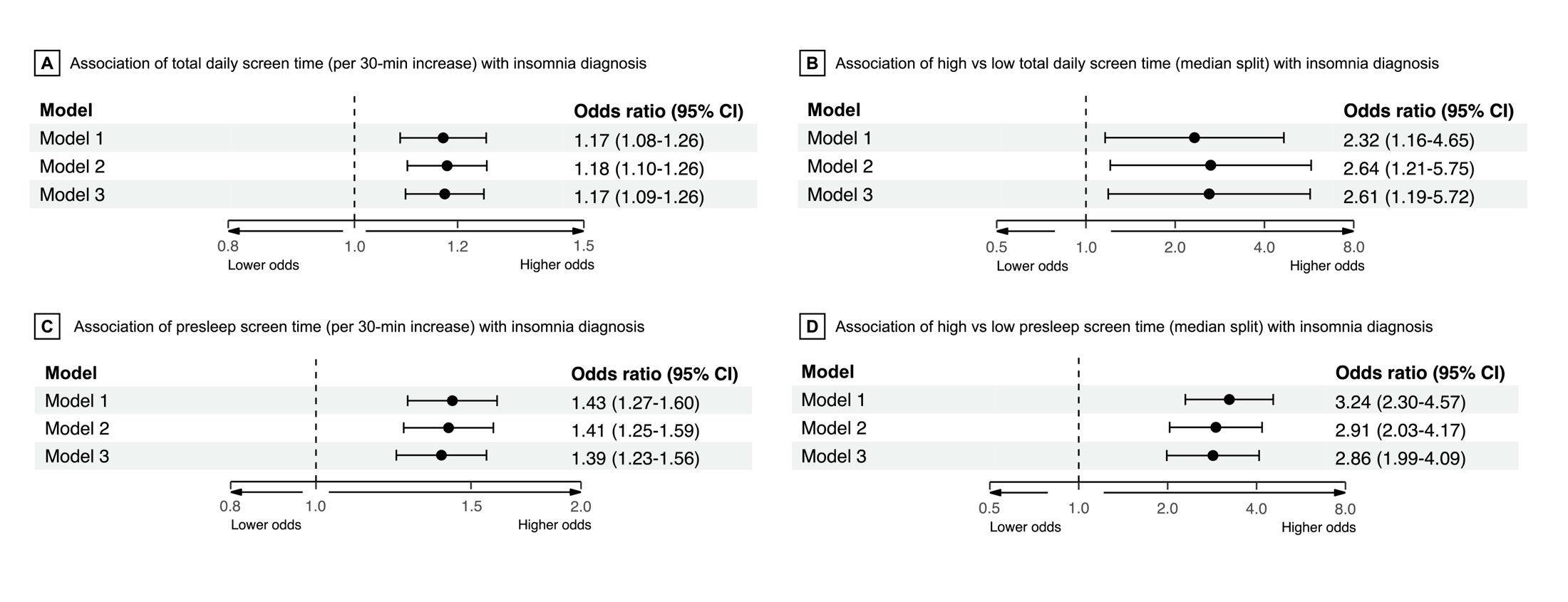
**

# **eTable 3** Sensitivity analysis for effect measure modification by perceived stigma on the association between total daily screen time and insomnia disorder with inverse probability treatment weighting

|  | **Low Screen Time** | |  | **High Screen Time** | | **OR (95% CI) for Insomnia with Strata of Perceived Stigma** |
| --- | --- | --- | --- | --- | --- | --- |
|  | N with/without Insomnia | OR (95% CI) |  | N with/without Insomnia | OR (95% CI) |  |
| **Low Perceived Stigma** | 49/18 | 1.0 (Reference) |  | 53/15 | 2.5 (0.92-6.75) | 2.5 (0.92-6.75) |
| **High Perceived Stigma** | 37/15 | 1.03 (0.38-2.82) |  | 47/6 | 2.87 (0.76-10.89) | 2.79 (0.74-10.52) |

Measure of effect measure modification on the additive scale: Relative Excess Risk due to Interaction (95% CI) = 0.35 (-3.41-4.1)

Measure of effect measure modification on the multiplicative scale: ratio of ORs (95% CI) = 1.12 (0.21-5.87)

ORs are adjusted for age, education level, profession, time since breast cancer diagnosis, TNM stage, current treatment, menopause status, alcohol consumption habits, and daily working hours by inverse probability treatment weighting.

# **eTable 4** Sensitivity analysis for effect measure modification by perceived stigma on the association between presleep screen time and insomnia disorder with inverse probability treatment weighting

|  | **Low Screen Time** | |  | **High Screen Time** | | **OR (95% CI) for Insomnia with Strata of Perceived Stigma** |
| --- | --- | --- | --- | --- | --- | --- |
|  | N with/without Insomnia | OR (95% CI) |  | N with/without Insomnia | OR (95% CI) |  |
| **Low Perceived Stigma** | 71/90 | 1.0 (Reference) |  | 120/40 | 3.46 (2.08-5.73) | 3.46 (2.08-5.73) |
| **High Perceived Stigma** | 101/63 | 2.08 (1.33-3.26) |  | 127/32 | 4.97 (2.98-8.3) | 2.39 (1.43-4) |

Measure of effect measure modification on the additive scale: Relative Excess Risk due to Interaction (95% CI) = 0.43 (-2.09-2.96)

Measure of effect measure modification on the multiplicative scale: ratio of ORs (95% CI) = 0.69 (0.34-1.42)

ORs are adjusted for age, education level, profession, time since breast cancer diagnosis, TNM stage, current treatment, menopause status, alcohol consumption habits, and daily working hours by inverse probability treatment weighting.

# **eFigure 3** Sensitivity Analysis for Association Between Total Daily Screen Time, Presleep Screen Time, and Insomnia Disorder with G-computation

**
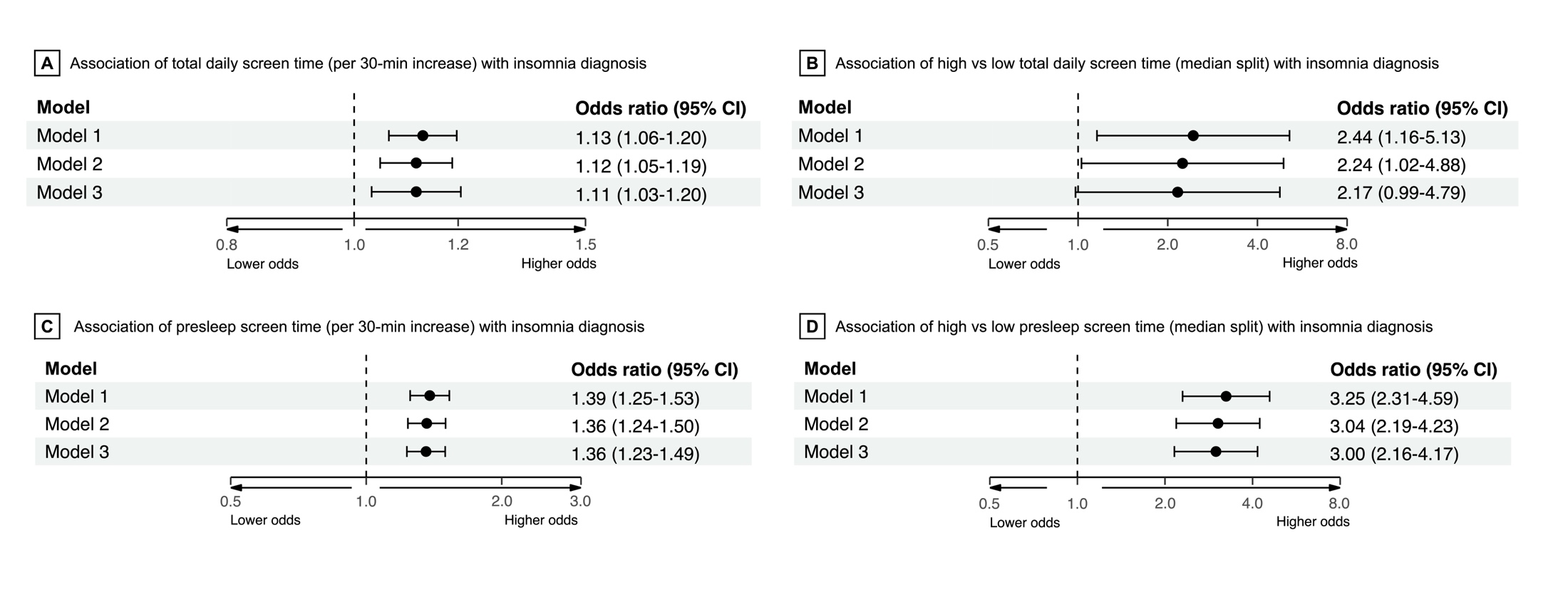
**

# **eTable 5** Sensitivity analysis for effect measure modification by perceived stigma on the association between total daily screen time and insomnia disorder with G-computation

|  | **Low Screen Time** | |  | **High Screen Time** | | **OR (95% CI) for Insomnia with Strata of Perceived Stigma** |
| --- | --- | --- | --- | --- | --- | --- |
|  | N with/without Insomnia | OR (95% CI) |  | N with/without Insomnia | OR (95% CI) |  |
| **Low Perceived Stigma** | 49/18 | 1.0 (Reference) |  | 53/15 | 1.61 (0.62-4.26) | 1.61 (0.62-4.26) |
| **High Perceived Stigma** | 37/15 | 1.06 (0.74-1.59) |  | 47/6 | 4.27 (1.26-16.37) | 4.04 (1.19-15.47) |

Measure of effect measure modification on the additive scale: Relative Excess Risk due to Interaction (95% CI) = 0.11 (-0.14-0.32)

Measure of effect measure modification on the multiplicative scale: ratio of ORs (95% CI) = 2.5 (0.56-11.64)

ORs are adjusted for age, education level, profession, time since breast cancer diagnosis, TNM stage, current treatment, menopause status, alcohol consumption habits, and daily working hours by G-computation.

# **eTable 6** Sensitivity Analysis for Effect Measure Modification by Perceived Stigma on the Association between Presleep Screen Time and Insomnia Disorder with G-computation

|  | **Low Screen Time** | |  | **High Screen Time** | | **OR (95% CI) for Insomnia with Strata of Perceived Stigma** |
| --- | --- | --- | --- | --- | --- | --- |
|  | N with/without Insomnia | OR (95% CI) |  | N with/without Insomnia | OR (95% CI) |  |
| **Low Perceived Stigma** | 71/90 | 1.0 (Reference) |  | 120/40 | 3.65 (2.36-5.79) | 3.65 (2.36-5.79) |
| **High Perceived Stigma** | 101/63 | 1.9 (1.33-2.84) |  | 127/32 | 4.58 (2.76-8.11) | 2.41 (1.46-4.28) |

Measure of effect measure modification on the additive scale: Relative Excess Risk due to Interaction (95% CI) = -0.12 (-0.26-0.03)

Measure of effect measure modification on the multiplicative scale: ratio of ORs (95% CI) = 0.66 (0.34-1.37)

ORs are adjusted for age, education level, profession, time since breast cancer diagnosis, TNM stage, current treatment, menopause status, alcohol consumption habits, and daily working hours by G-computation.

# **eFigure 4** Sensitivity Analysis for Association Between Presleep Screen Time, and Insomnia Disorder with Median/Mode Imputation ^a^


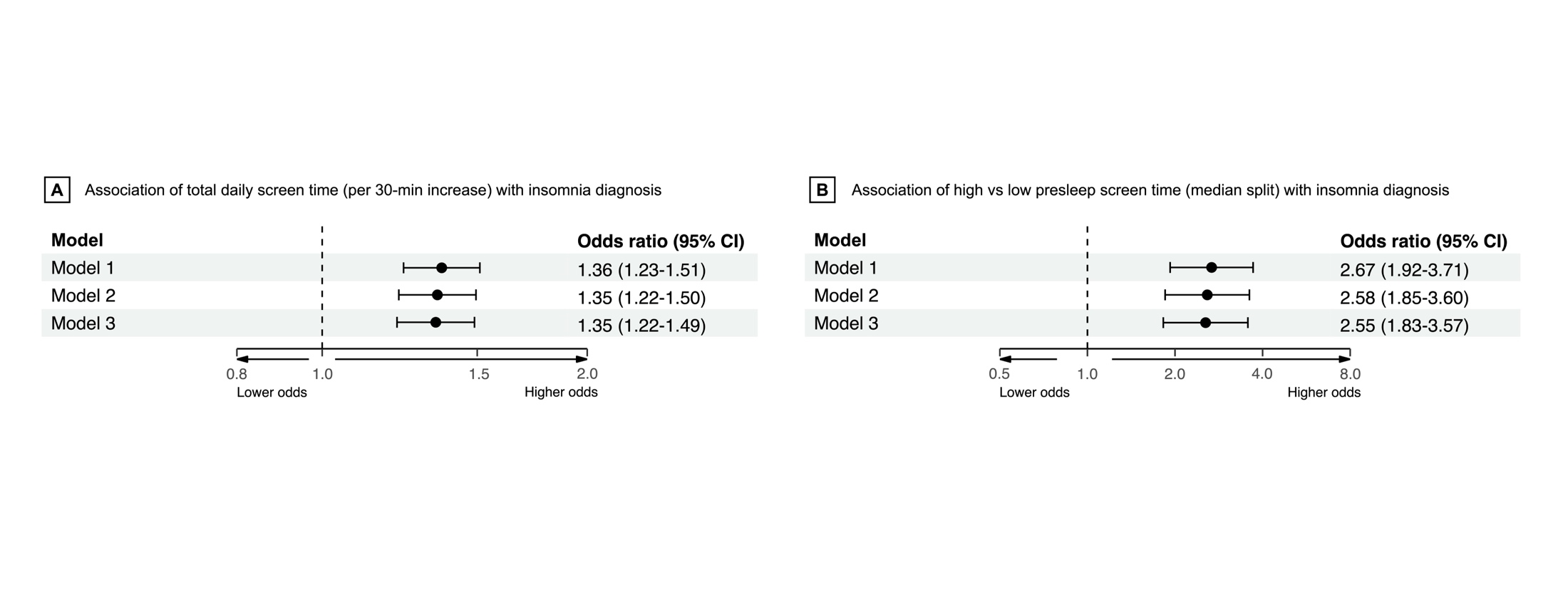


a. Categorical variables were imputed using mode imputation and continuous variables using median imputation.

# **eTable 7** Sensitivity Analysis for Effect Measure Modification by Perceived Stigma on the Association between Presleep Screen Time and Insomnia Disorder with Median/Mode Imputation ^a^

|  | **Low Screen Time** | |  | **High Screen Time** | | **OR (95% CI) for Insomnia with Strata of Perceived Stigma** |
| --- | --- | --- | --- | --- | --- | --- |
|  | N with/without Insomnia | OR (95% CI) |  | N with/without Insomnia | OR (95% CI) |  |
| **Low Perceived Stigma** | 87/98 | 1.0 (Reference) |  | 124/47 | 3 (1.9-4.75) | 3 (1.9-4.75) |
| **High Perceived Stigma** | 119/68 | 1.9 (1.23-2.92) |  | 133/36 | 4.12 (2.54-6.71) | 2.17 (1.33-3.56) |

a. Categorical variables were imputed using mode imputation and continuous variables using median imputation.

Measure of effect measure modification on the additive scale: Relative Excess Risk due to Interaction (95% CI) = 0.22 (-1.77-2.22)

Measure of effect measure modification on the multiplicative scale: ratio of ORs (95% CI) = 0.72 (0.37-1.42)

ORs are adjusted for age, education level, profession, time since breast cancer diagnosis, TNM stage, current treatment, menopause status, alcohol consumption habits, and daily working hours by inverse probability treatment weighting.

# **eFigure 5** Sensitivity analysis for association between total daily screen time, presleep screen time, and insomnia severity

**
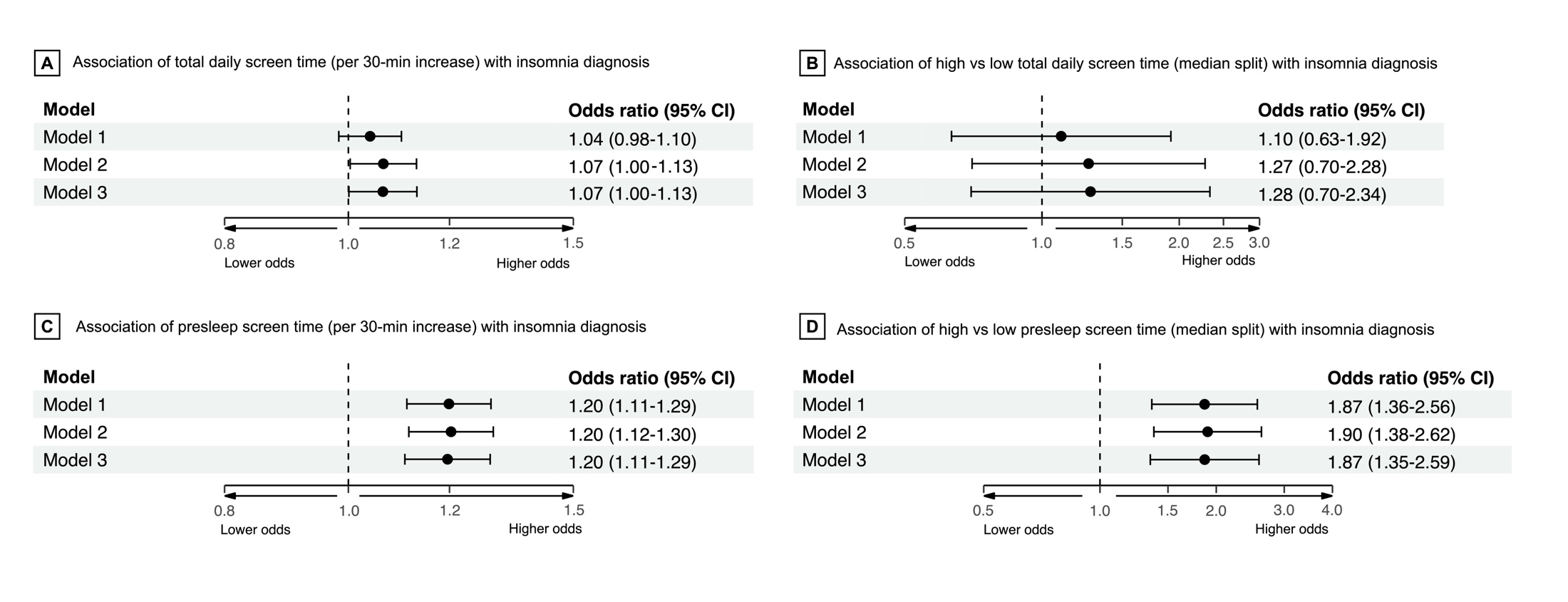
**

# **eTable 8** Sensitivity analysis for effect measure modification by perceived stigma on the association between total daily screen time and insomnia severity

|  | **Low Screen Time** | |  | **High Screen Time** | | **OR (95% CI) for More Severe Insomnia with Strata of Perceived Stigma** |
| --- | --- | --- | --- | --- | --- | --- |
|  | N with/without Insomnia ^a^ | OR (95% CI) |  | N with/without Insomnia | OR (95% CI) |  |
| **Low Perceived Stigma** | 29/38 | 1.0 (Reference) |  | 29/39 | 1.02 (0.47-2.22) | 1.02 (0.47-2.22) |
| **High Perceived Stigma** | 25/27 | 0.98 (0.46-2.1) |  | 24/29 | 1.68 (0.74-3.8) | 1.72 (0.73-4.03) |

a. Insomnia severity was measured using the Insomnia Severity Index (ISI) with categories 0=none, 1=mild, 2=moderate, 3=severe; for cross-tabulation purposes, participants were classified as having insomnia symptoms (ISI categories 1-3) versus no insomnia symptoms (ISI category 0).

Measure of effect measure modification on the additive scale: Relative Excess Risk due to Interaction (95% CI) = 0.68 (-0.57-1.93)

Measure of effect measure modification on the multiplicative scale: ratio of ORs (95% CI) = 1.68 (0.57-4.96)

ORs are adjusted for age, education level, profession, time since breast cancer diagnosis, TNM stage, current treatment, menopause status, alcohol consumption habits, and daily working hours.

# **eTable 9** Sensitivity analysis for effect measure modification by perceived stigma on the association between presleep screen time and insomnia severity

|  | **Low Screen Time** | |  | **High Screen Time** | | **OR (95% CI) for More Severe Insomnia with Strata of Perceived Stigma** |
| --- | --- | --- | --- | --- | --- | --- |
|  | N with/without Insomnia ^a^ | OR (95% CI) |  | N with/without Insomnia | OR (95% CI) |  |
| **Low Perceived Stigma** | 44/117 | 1.0 (Reference) |  | 60/100 | 1.49 (0.92-2.41) | 1.49 (0.92-2.41) |
| **High Perceived Stigma** | 63/101 | 1.46 (0.91-2.36) |  | 89/70 | 3.45 (2.16-5.53) | 2.36 (1.52-3.68) |

a. Insomnia severity was measured using the Insomnia Severity Index (ISI) with categories 0=none, 1=mild, 2=moderate, 3=severe; for cross-tabulation purposes, participants were classified as having insomnia symptoms (ISI categories 1-3) versus no insomnia symptoms (ISI category 0).

Measure of effect measure modification on the additive scale: Relative Excess Risk due to Interaction (95% CI) = 1.5 (0.24-2.77)

Measure of effect measure modification on the multiplicative scale: ratio of ORs (95% CI) = 1.59 (0.83-3.05)

ORs are adjusted for age, education level, profession, time since breast cancer diagnosis, TNM stage, current treatment, menopause status, alcohol consumption habits, and daily working hours.

# **eFigure 6** Sensitivity analysis for association between total daily screen time, presleep screen time, and sleep quality


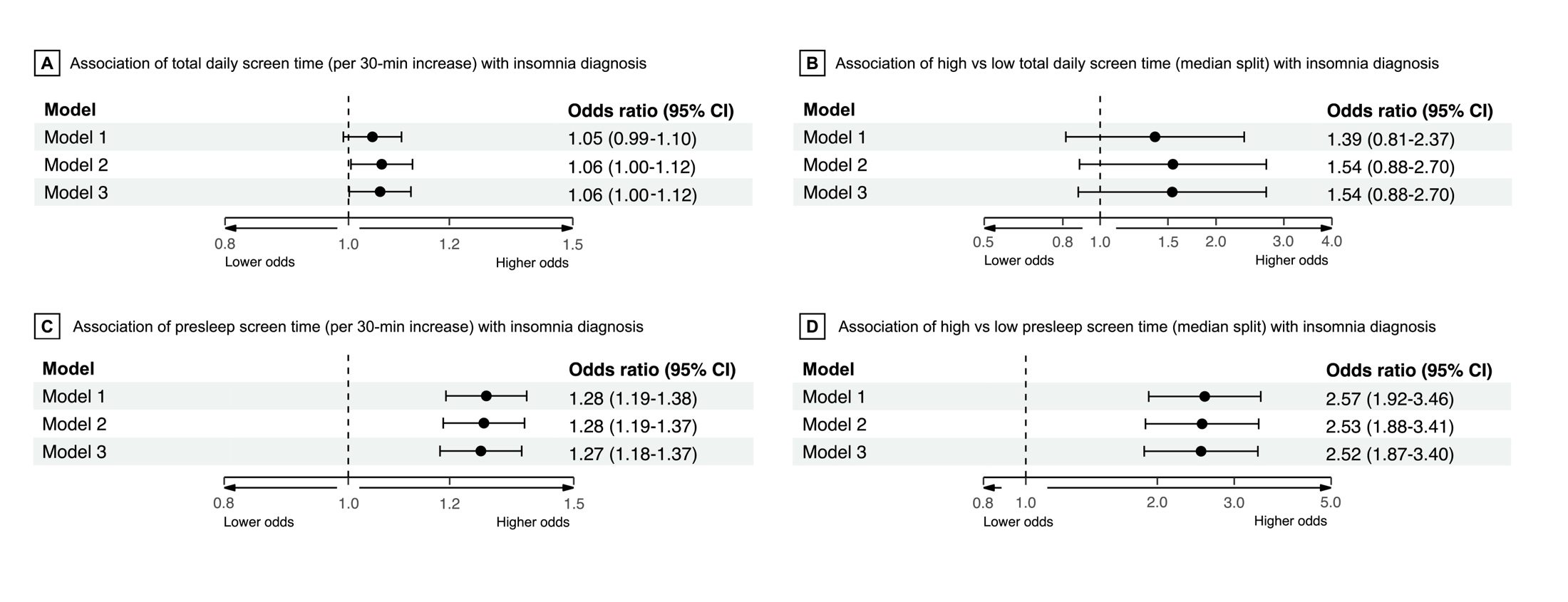


# **eTable 10** Sensitivity analysis for effect measure modification by perceived stigma on the association between total daily screen time and sleep quality

|  | **Low Screen Time** | |  | **High Screen Time** | | **OR (95% CI) for Poorer Sleep Quality with Strata of Perceived Stigma** |
| --- | --- | --- | --- | --- | --- | --- |
|  | N poor/good sleep quality ^a^ | OR (95% CI) |  | N poor/good sleep quality | OR (95% CI) |  |
| **Low Perceived Stigma** | 20/47 | 1.0 (Reference) |  | 19/49 | 1.1 (0.53-2.27) | 1.1 (0.53-2.27) |
| **High Perceived Stigma** | 15/37 | 1.31 (0.65-2.62) |  | 18/35 | 2.99 (1.39-6.42) | 2.28 (1.05-4.93) |

a. Sleep quality was measured using the Pittsburgh Sleep Quality Index (PSQI) with categories 0=very good, 1=good, 2=fair, 3=poor; for cross-tabulation purposes, participants were classified as having poor sleep quality (PSQI categories 2-3) versus good sleep quality (PSQI categories 0-1).

Measure of effect measure modification on the additive scale: Relative Excess Risk due to Interaction (95% CI) = 1.58 (-0.29-3.45)

Measure of effect measure modification on the multiplicative scale: ratio of ORs (95% CI) = 2.08 (0.78-5.57)

ORs are adjusted for age, education level, profession, time since breast cancer diagnosis, TNM stage, current treatment, menopause status, alcohol consumption habits, and daily working hours.

# **eTable 11** Sensitivity analysis for effect measure modification by perceived stigma on the association between presleep screen time and sleep quality

|  | **Low Screen Time** | |  | **High Screen Time** | | **OR (95% CI) for Poorer Sleep Quality with Strata of Perceived Stigma** |
| --- | --- | --- | --- | --- | --- | --- |
|  | N poor/good sleep quality ^a^ | OR (95% CI) |  | N poor/good sleep quality | OR (95% CI) |  |
| **Low Perceived Stigma** | 30/131 | 1.0 (Reference) |  | 46/114 | 2.73 (1.77-4.21) | 2.73 (1.77-4.21) |
| **High Perceived Stigma** | 33/131 | 1.78 (1.16-2.74) |  | 60/99 | 4.35 (2.81-6.73) | 2.44 (1.62-3.68) |

a. Sleep quality was measured using the Pittsburgh Sleep Quality Index (PSQI) with categories 0=very good, 1=good, 2=fair, 3=poor; for cross-tabulation purposes, participants were classified as having poor sleep quality (PSQI categories 2-3) versus good sleep quality (PSQI categories 0-1).

Measure of effect measure modification on the additive scale: Relative Excess Risk due to Interaction (95% CI) = 0.84 (-0.74-2.42)

Measure of effect measure modification on the multiplicative scale: ratio of ORs (95% CI) = 0.89 (0.49-1.62)

ORs are adjusted for age, education level, profession, time since breast cancer diagnosis, TNM stage, current treatment, menopause status, alcohol consumption habits, and daily working hours.
